# How does digital transformation affect corporate sustainability performance? Evidence from listed energy companies in China

**DOI:** 10.1371/journal.pone.0325898

**Published:** 2025-06-13

**Authors:** Li Guo, Fangxia Chen, Linhao Chen

**Affiliations:** 1 Energy Economy and Management Research Center, Xi’an University of Science and Technology, Xi’an, China; 2 School of Management, Xi’an University of Science and Technology, Xi’an, China; 3 School of Economics and Management, Xi’an Shiyou University, Xi’an, China; Peking University, CHINA

## Abstract

Promoting sustainable growth in the energy sector is key to China’s high-quality development under socialist modernization. Based on dynamic capability theory and the resource-based view, this study uses unbalanced panel data (2011–2023) of Chinese listed energy enterprises to examine how digital transformation (DT) impacts sustainable development performance (SDP), including its subdimensions: financial performance, environmental‒social governance (ESG), and innovation. Using two-way fixed effects models and mediation analysis, we find that (1) DT significantly enhances SDP; (2) this improvement operates through alleviating financing constraints and optimizing resource allocation efficiency; and (3) the benefits are more pronounced in small-scale enterprises and downstream segments of the energy value chain. These findings deepen the understanding of the role of DTs in sustainable development and provide practical guidance for energy firms.

## 1. Introduction

Over the previous ten years, the average yearly growth rate has been approximately 3.2%, and China is among the world’s largest energy consumers. It is anticipated that China will need 5.72 billion tonns of regular coal annually in 2023. China’s economic growth is driven primarily by energy. Since businesses are an essential part of the economy and society, they are the main source of pollution emissions and pollution control [1], and strong economic growth is closely tied to their sustainable development. Therefore, how to protect the environment while pursuing economic benefits has become a significant problem that energy companies must resolve. In addition to being a necessary component of excellent economic development, assisting energy companies in achieving sustainable development is a crucial step in addressing environmental change. Enterprise sustainable development performance, which is the total result of economic, environmental, social, and innovative performance [2], is a statistic used to evaluate how sustainably firms are developing [3]. How to accomplish the sustainable development of energy companies has emerged as a significant challenge for businesses, as the notion of sustainable development has gradually gained depth.

The 20th Party Congress report emphasized the need to accelerate the growth of the digital economy. As the internet economy has expanded, digital enterprises are important and cannot be ignored; only by vigorously developing and supporting businesses’ digital transformation can we foster the digital economy’s rapid and healthy growth. Blockchain, artificial intelligence, and other cutting-edge technologies are all part of the high-level shift of digital transformation. It also modifies and enhances the conventional business models [4]. Digital transformation has been shown to improve the economic, environmental, and social benefits of firms [5].

Some scholars have also reported that digital transformation indirectly promotes enterprises’ green technology innovation performance by increasing R&D investment, optimizing human capital structure and improving management efficiency [6]; it also promotes significant enterprise green innovation efficiency in a low-carbon context [7].

However, according to some academics, digital technology is not necessarily conducive to the stability of the overall enterprise system, as it increases enterprise management costs and reduces financial performance and R&D investment [8]. Data storage and transmission consume large amounts of energy [9], increasing carbon emissions and resource consumption, which consequently has a detrimental effect on corporate environmental performance. Thus, more research is needed to determine whether digital transformation can improve energy companies’ sustainability performance.

This study uses panel data from A-share listed energy companies in Shanghai and Shenzhen (2011–2023). Using Python-based techniques to generate corporate digital transformation indices, this study investigates the implications of digital transformation for firms’ sustainable development performance from both theoretical and empirical perspectives. Corresponding recommendations are also made. The potential contributions of this paper are as follows:

(1) The entropy method is used to assess the performance of sustainable development in three dimensions—financial, environmental/social governance (ESG), and innovation—to examine how the digital revolution impacts energy businesses’ sustainable development performance, thereby promoting the study of sustainable development performance and digital transformation.
(2) The pathway framework for enhancing the success of energy companies in sustainable development is supplemented by revealing how digital transformation influences the sustainable development performance of businesses from the viewpoints of financing constraints and resource allocation efficiency.
(3) It examines the diverse features of businesses’ digital transformation to advance their sustainable development performance from the viewpoints of business size and the energy value chain of energy companies.

## 2. Theoretical analysis and research hypotheses

### 2.1. Digital transformation and the sustainable performance of energy companies

Data assets and digital technology applications are increasingly crucial parts of strategic resources, and the composition of resources that businesses rely on has changed significantly due to digital transformation. These digital resources provide companies with a new competitive foundation to shape their competitive position. The resource-based view emphasizes the endogenous nature of enterprise development, arguing that enterprise growth and development rely on its internal resource capabilities [10]. Digital technologies enable enterprises to identify resource needs and expand the scope of their resource acquisition, laying the foundation for sustainable growth.

From the standpoint of financial results, first, digital enterprises effectively reduce their cost expenditures, improving financial performance by using cutting-edge digital tools and technologies to reshape their company processes. The application of automated processes and intelligent systems reduces the error rate from human intervention, lowering product defect and scrap rates and, thus, reducing the cost of scrap disposal and reprocessing [11]. Through digital solutions, companies are better equipped to analyze and forecast market demand accurately, avoid overproduction and inventory backlogs, and further reduce inventory costs [12]. Second, companies improve their profitability by using technological tools, such as computers, big data and the internet. With the support of digitalization, service-oriented modular production extends the value chain’s value-added space, provides customers with a self-service business model intervention window, more accurately aligns market demand and enterprise development goals, and is conducive to enhancing operational efficiency and profitability [13]. Digital technology may be utilized by businesses to develop new business, realize business model innovation, expand value creation channels, and improve sales revenue [14]. Finally, firms improve their operational efficiency by investing in digital technologies and using budgeting software and big data analytics techniques to enhance decision-making and streamline processes [15]. Digital tools and platforms make it easier to exchange information between enterprise departments, avoiding the problem of data fragmentation [16,17].

Digital transformation enhances environmental, social, and governance performance through three primary mechanisms. First, it enables businesses to optimize product design, development workflows, and resource utilization while monitoring and controlling pollution and energy consumption during manufacturing processes. By leveraging intelligent digital technologies, companies can significantly improve their environmental performance [18]. Second, digital transformation fosters open, collaborative, and data-driven decision-making frameworks. Through digital platforms, enterprises facilitate stakeholder engagement, strengthen strategic alignment with social responsibility objectives, and enhance responsiveness to diverse stakeholder demands during strategic planning [19]. Finally, organizational digitalization promotes information transparency and interdepartmental connectivity, reducing fragmented governance structures and enabling the establishment of efficient, cross-functional collaboration models [20].

Digital transformation enhances innovation performance through four key pathways. First, organizations leverage digitalization to accelerate product and operational innovations through optimized internal resource integration and cross-functional collaboration [21]. Second, cost efficiencies generated by digital adoption free up capital for R&D investment, enabling focused development of novel products/services that boost market competitiveness. Third, digitalization restructures innovation processes by embedding multistakeholder value chains into digitally connected systems—from ideation and product development to manufacturing and distribution—compressing innovation cycle durations by 42–58% while reducing costs [22]. Digital transformation alters the original innovation process. Businesses can utilize digital technology to embed the main body of multivalue links in the innovation chain, compressing the innovation results of time and cost transformations, thereby improving innovation performance [23]. The intelligent transformation of enterprises significantly improves the quantity and quality of green innovation [24] and promotes the technological innovation of new energy enterprises [25]. Digitally mature organizations cultivate innovation-centric cultures that institutionalize experimentation and organizational learning, creating adaptive frameworks for rapid responses to technological and market disruptions [26].

In summary, energy firms’ sustainability performance can be enhanced via digital transformation. Thus, the following theories are proposed in this study:

H1: Digital transformation positively impacts energy firms’ sustainable development performance.

H1a: Energy companies’ financial performance benefits from digital transformation.

H1b: The environmental and social governance performance of energy companies is positively impacted by digital transformation.

H1c: The inventive performance of energy firms is positively impacted by digital transformation.

### 2.2. The mediating role of financial constraints

Digital transformation addresses financial constraints through three strategic mechanisms. First, capital market digitization accelerates transactional efficiency across issuance, listing, and settlement processes, reducing operational costs by 18–25% while enhancing funding accessibility [27,28]. Second, enhanced digital governance improves information transparency, lowering enterprise risk premiums by 1.2–2.4 percentage points in external financing evaluations [29]. Third, blockchain-enabled supply chain integration reduces information asymmetry between upstream/downstream partners by 37–41%, boosting investor confidence in innovation pipelines and securing R&D funding [30].

Financing constraints will impact businesses’ ability to develop sustainably. First, energy enterprise characteristics, such as high investment, high risk and limited financing channels, impose strong financing constraints on conducting their business activities, preventing them from maximizing profit and improving financial performance, which makes it challenging to maintain an ideal capital structure [31]. Second, alleviating financing constraints provides private enterprises with the necessary resources to improve ESG performance, enabling them to provide more financial support for green production, and, thus, improve ESG performance [32]. Finally, the easing of financing constraints enables retail enterprises to undertake larger-scale R&D investments and introduce more sophisticated tools and technologies. The increase in these inputs improves the capacity for invention and market competitiveness, driving enterprises to achieve more breakthroughs in product innovation, service innovation, marketing innovation, etc. [33].

In summary, digital transformation promotes energy companies’ sustainability performance by removing financial restrictions. Thus, this study proposes the following hypotheses:

H2: Financial constraints act as a mediator in the interaction between energy companies’ sustainable development performance and digital transformation.

H2a: Budgetary limitations serve as moderators in the interaction between energy companies’ financial success and digital transformation.

H2b: Financial constraints act as a mediating factor in the connection between energy businesses’ social and environmental governance performance and digital transformation.

H2c: The relationship between energy organizations’ creative performance and digital transformation is mediated by financial limitations.

### 2.3. Mediating role of resource allocation efficiency

Differences in dynamic capabilities can lead to differences in the performance of firms with homogeneous resource bases [34]. The key to energy companies’ long-term success lies in maximizing output through the rational allocation of resources, the efficiency of which is crucial. The primary methods of digital transformation to increase energy companies’ resource allocation efficiency include the following: First, digital technology can quickly obtain more effective information, which allows identifying more opportunities to increase resource allocation efficiency [35]. Second, digital technology promotes technical exchange among enterprises and provides internal transformation momentum to increase the effectiveness of enterprises’ resource allocation [36]. Third, businesses use digital tools, such as big data and blockchain, to connect with customers promptly, improve communication efficiency and acquire more customer resources, thereby improving the allocation of current resources and reducing reliance on leading clients [37].

The greater the resource allocation efficiency, the more effectively energy businesses can improve their sustainable development performance. First, improving resource allocation efficiency can reduce the probability of over- and underinvestment, investment efficiency can affect enterprise profitability, and better business and financial performance are directly correlated with increased investment efficiency [38]. Second, digital attention enables enterprise decision-makers to rationally allocate resources and enhances the effectiveness of resource distribution, improving enterprise ESG performance [39]. Finally, enterprises apply digital technology to optimize the distribution of traditional labor, increase the amount of nontraditional employment, increase R&D spending, invest more in digital infrastructure, and strengthen their digital innovation capabilities [40].

In summary, digital transformation facilitates the sustainable development performance of energy firms by enhancing resource allocation efficiency. Thus, this study proposes the following hypotheses:

H3: Resource allocation efficiency acts as a mediator between the sustainable development performance of energy-related businesses and digital transformation.

H3a: Resource allocation efficiency acts as a mediator between energy companies’ financial performance and digital transformation.

H3b: Digital transformation and energy businesses’ social and environmental governance performance are mediated by resource allocation efficiency.

H3c: The innovative performance of energy corporations and digital transformation are mediated by resource allocation efficiency.

**Fig 1:**
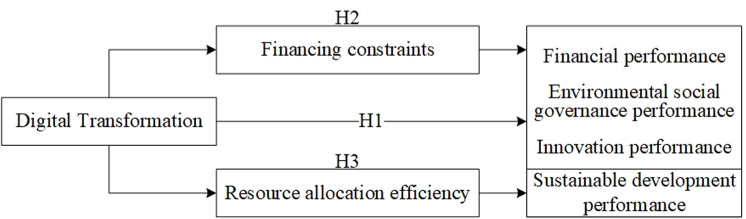
Theoretical model.

In summary, this paper constructs a theoretical model, which is shown in Fig 1.

## 3. Study design

### 3.1. Data and sample

The criteria of the Guidelines for Environmental Information Disclosure of Listed Companies indicate that the operating conditions and development of energy firms significantly impact China’s energy structure and sustainable development. Thus, to investigate experimentally how digital transformation affects energy organizations’ sustainable development performance, this study uses data from A-share-listed energy enterprises from 2011 to 2023 as the sample. The energy industry types chosen for this article explicitly include the following, after the China Securities Regulatory Commission updated the Guidelines for Industry Classification of Listed Companies in 2012: coal mining and washing (B06), oil and gas extraction (B07), mining auxiliary activities (B11), coking, nuclear fuel processing, and petroleum processing (C25), the manufacturing and distribution of electric power and heat (D44), gas production and supply (D45), and the production and supply of water (D46).

This study employed Stata 17.0 for data processing and empirical analyses. To avoid meaningless data from interference, the following data and research findings were processed: (1) ST and ST* enterprises were excluded; (2) enterprises listed for less than three years were excluded; and (3) enterprises with seriously missing data were excluded. Ultimately, 149 enterprises were excluded, resulting in 1,465 legitimate observations in total. To lessen the effect of extreme values, the main continuous variables were winsorized at 1% to mitigate extreme values.

The data sources of this paper are as follows: (1) Financial performance (ROA) is derived from the CSMAR database; ESG scores are derived from the composite scores of ESG disclosure published by the CSI; and patent application data are derived from the China Research Data Service Platform (CNRDS) and the patent search system of the State Intellectual Property Office. (2) Digital transformation data are obtained from the annual reports of listed companies on the Juchao Information Network, which are analyzed and organized by Python technology. (3) The KZ index is obtained from Cathay Pacific’s database; and the relevant data required for TFP measurement are obtained from the China Urban Statistical Yearbook, China Environmental Statistical Yearbook, social responsibility reports of listed companies and annual reports, etc. The other financial data of enterprises are obtained from the CNRDS and the patent search system of the State Intellectual Property Office. All other corporate financial data are from the CSMAR and Wind databases.

### 3.2. Selection of variables

#### 3.2.1. Explained variables.

Sustainable development performance. The performance of sustainable development is defined as an enterprise’s ability to reduce costs, improve efficiency, and reduce waste, pollution, and energy use to support its growth in economic social, and environmental sustainable development [41]. This study draws on the methods of Ameer [42] and Chen [43]. This study assesses corporate sustainability performance in three subdimensions: financial, environmental–social governance, and innovation performance. When combined with the traits of science and technology, innovation contributes significantly to the sustainable energy development of energy firms. ① Financial performance (ROA), typically measured by the ratio of return on net assets to total return on assets, is used. ROA is used to measure the short-term operating efficiency of the firm and Tobin’s Q value (Tobinq) is used to measure the future value of the enterprise. Considering market changes, return on assets (ROA) is used to gauge the financial performance of corporations [44]. ② Environmental–social governance performance (ESG) scores are used to gauge corporate environmental and social governance performance, which is indicated by a higher score. ESG data are mainly from third-party organizations (e.g., CSI Shangdao RongLiGreen, Bloomberg). Since 2009, the CSI ESG index has been used to assess securities issuers’ ESG performance, such as A-shares and debt issuers, and has received widespread recognition from both academia and industry [45] for providing scientifically validated ratings. Other ESG rating systems cannot cover most companies, and their data time horizons are narrow [46]. Thus, this paper adopts the environmental score of the CSI ESG rating system to measure corporate environmental performance. ③ For innovation performance (EIP), the number of patent applications serves as a robust indicator of innovation capacity [47, 48]. This metric accurately reflects an organization’s true innovative capability. ④ Sustainable development performance (SDP) is calculated using the complete score of the entropy value technique based on the aforementioned indicators, assessing how well energy companies operate in terms of sustainable development [49].

#### 3.2.2. Explanatory variable.

**Fig 2:**
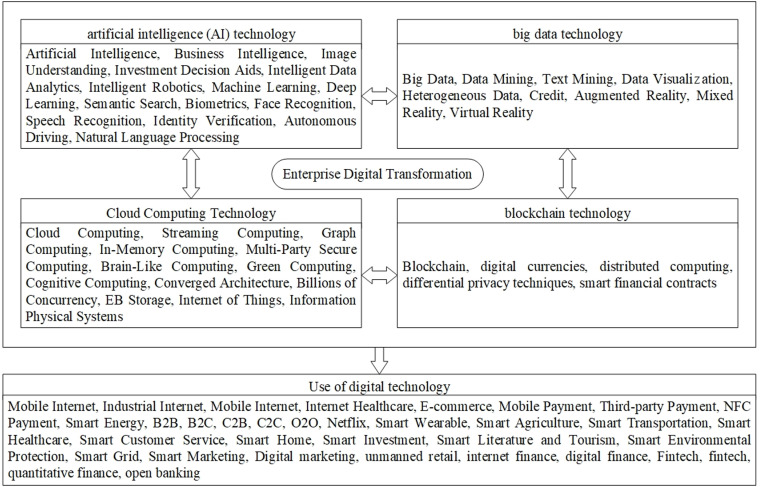
Structured feature lexicon for enterprise digital transformation.

Digital transformation (DCG). This study quantifies digital transformation through the text mining of corporate annual reports. First, Python-based web scraping was employed to collect 2011–2023 annual reports of A-share listed energy firms from official websites, followed by text conversion. Second, there are two aspects of digital transformation, namely, the application of underlying technology and how it is used in practice, and keywords, as indicated in Fig 2. Third, Python technology was used to extract keywords associated with digital transformation from annual reports for businesses, resulting in 76 keywords in two categories—the use of underlying technology and the practical use of technology—to build the index system for businesses’ digital transformation [50]. To address right-skewed distributions, a ln(x + 1) transformation was applied to the raw keyword frequencies. Additionally, this study discusses Li’s practice of enriching the digital transformation keyword thesaurus, using Python technology to crawl 139 keyword word frequency sums, and constructing digital transformation indicators to perform robustness tests [51].

#### 3.2.3. Mediating variables.

(1) The financing constraint (KZ) index is obtained through an ordered logistic regression with the Tobin’s Q value, gearing ratio, dividend payout ratio, cash holding ratio, and enterprise operating cash flow as independent factors. The larger the KZ index, the greater the financial distress and financing constraints the firm faces. Therefore, the KZ index is used as a proxy variable in this study. ② Resource allocation efficiency (TFP) is measured by scholars mainly using parametric and semiparametric methods. This study measures the resource allocation efficiency of heavily polluting enterprises using the semiparametric approach and the total productivity of businesses via the LP method [52, 53].

#### 3.2.4. Control variables.

Drawing on the research of relevant scholars, this paper considers enterprise size (Size), total asset turnover (ATO), equity concentration (TOP10), enterprise growth (GRO), the independent director ratio (Indep), two positions in one (Dual), checks and balances for equity (Balance), the size of the board of directors (Board), government subsidies (GS), audit costs (AuditFee), and employee size (Employee). The board of directors’ size (Board), government subsidies (GS), audit fees (AuditFee), and employee size (Employee) were used as control variables [54, 55]. Table 1 displays the complete explanations and definitions for every variable used in this work.

**Table 1:** Variable definitions and descriptions.

Variable Type	Variable Name	Variable Symbol	Definition Description
Explained variables	Financial performance	ROA	Total assets/net profit at year’s end
	Environmental social governance performance	ESG	CSI ESG Composite Score
	Innovation performance	EIP	(Number of patent applications in the period + 1) in natural logarithms
	Sustainable development performance	SDP	Indexes obtained from entropy synthesis
Explanatory variable	Digital transformation	DCG	(Total number of enterprise digital transformation word frequencies + 1) taken as a natural logarithm
Mediating variables	Financing constraints	KZ	KZ index
	Resource allocation efficiency	TFP	Total productivity of enterprises based on the LP methodology
Control variables	enterprise size	Size	Total assets taken as a natural logarithm
	total asset turnover	ATO	Operating income/average total assets
	equity concentration	TOP10	The proportion of shares held by the company’s top ten shareholders
	enterprise growth	GRO	Operating income as of right now compared to operating income in the past
	independent director ratio	Indep	Number of independent directors/directors
	two positions in one	Dual	Chairman and general manager of the two positions together assigned to 1, otherwise 0
	equity checks and balances	Balance	The percentage of shares owned by the first-largest shareholder compared to the second-largest shareholding
	board of directors’ size	Board	A natural logarithm is used to calculate the number of board members
	government subsidy	GS	(Amount of government grants for the period + 1) in natural logarithms
	audit fee	AuditFee	Audit costs are expressed in natural logarithms
	employee size	Employee	The number of employees is taken as a natural logarithm

### 3.3. Model Construction

Given the use of panel data in this analysis, a two-way fixed effects model was adopted to mitigate potential issues, such as omitted variable bias in the model design. The following multiple linear regression framework was constructed to test the hypotheses:


$$\begin{array}{c} ROA_{i,t}\;/ESG_{i,t}\;/EIP_{i,t}\;/SDP_{i,t}\;=\;\alpha_{0}+\;\alpha_{1}DCG_{i,t}\;+\alpha_{2}Controli,t\;+\;Ind_{i}\;+\;Year_{t}\;+\;\varepsilon_{i,t} \end{array}$$ (1)

In Equation (1), *i* represents firms, *t* represents years, ROA_*i,t*_, ESG_*i,t*_, EIP_*i,t*_ and SDP_*i,t*_ represent the financial performance, ESG performance, innovation performance and sustainability performance of firm *i* in year *t*, respectively, and they are all explained variables; DCG_*i,t*_ is the main explanatory factor, which shows the degree of digital transformation of firm *i*’s enterprise in year *t*; Control includes several control factors that may affect the sustainability effects; *Ind* and *Year* represent industry fixed effects and year fixed effects, respectively; and *ε*_*i,t*_ is a random perturbation term. If H1 is proven correct, the coefficient *α*_*1*_ is significantly positive.

## 4. Empirical analyses

### 4.1. Descriptive statistics

Table 2 presents the descriptive statistics for all variables. The substantial range between the maximum and minimum values of energy corporations’ financial performance (ROA), environmental–social governance performance (ESG), innovation performance (EIP), and sustainable development performance (SDP) reflects significant heterogeneity in sustainable development outcomes across Chinese energy firms. The mean SDP (0.436) slightly exceeds the median (0.436), with over 50% of observations below the mean, indicating room for improvement in China’s energy sector sustainability. Digital transformation (DCG) exhibits extreme divergence (max = 2.890, min = 0), highlighting uneven adoption levels. The comparable mean (0.691) and median (0.693) DCG values suggest that half of the firms remain at average digitalization levels, underscoring the need for broader technological advancement. The control variables demonstrate reasonable dispersion ranges.

**Table 2:** Results of descriptive statistics.

Variable	Obs	Mean	Std	Max	Median	Min
ROA	1,465	0.030	0.051	0.165	0.031	−0.176
ESG	1,465	73.197	5.283	84.100	73.170	57.250
EIP	1,465	2.750	1.961	7.670	2.833	0.000
SDP	1,465	0.436	0.126	0.753	0.433	0.114
DCG	1,465	0.691	0.821	2.890	0.693	0.000
KZ	1,465	1.559	1.987	6.270	1.758	−3.865
TFP	1,465	8.635	1.086	11.068	8.631	6.099
Size	1,465	23.415	1.646	28.036	23.359	19.634
ATO	1,465	0.528	0.428	2.632	0.417	0.065
TOP10	1,465	0.643	0.172	0.958	0.645	0.221
GRO	1,465	0.141	0.405	2.591	0.071	−0.436
Indep	1,465	36.749	4.464	50.000	35.290	33.330
Dual	1,465	0.102	0.303	1.000	0.000	0.000
Balance	1,465	0.319	0.286	0.967	0.243	0.008
Board	1,465	2.236	0.227	2.708	2.197	1.609
GS	1,465	16.087	3.281	20.890	16.595	0.000
AuditFee	1,465	14.275	0.947	17.674	14.116	12.612
Employee	1,465	8.346	1.550	12.837	8.196	4.248

### 4.2. Benchmark regression analyses

Model (1) evaluates the impact of digital transformation (DT) on the sustainable development of energy enterprises; the statistical findings are reported in Table 3. In Table 3, Columns (1) through (4) present the results without control variables, whereas Columns (5) to (6) present the outcomes with control variables added. The empirical findings underscore that the coefficient *α*_*1*_ for DCG remains significantly positive regardless of the inclusion of sustainability performance control variables and their three component subdimensions. As shown in Column (5), the DCG coefficient is 0.005, which is significantly positive at the 1% level, validating Hypothesis H1a and indicating that digital transformation has enhanced the financial performance of energy businesses. Column (6) shows a DCG coefficient of 0.399, which is significantly positive at the 5% level, verifying Hypothesis H1b. This suggests that increased digital transformation in energy companies improves their environmental and social governance performance. Column (7) shows a DCG coefficient of 0.089, which is significantly positive at the 5% level, supporting Hypothesis H1c. This implies that greater digital transformation in energy businesses enhances their innovation performance. Column (8) reveals a DCG coefficient of 0.009, which is significantly positive at the 1% level, confirming Hypothesis H1. This finding indicates that higher levels of digital transformation in energy businesses lead to improved sustainability performance.

**Table 3:** Benchmark regression results.

Variable	(1)	(2)	(3)	(4)	(5)	(6)	(7)	(8)
ROA	ESG	EIP	SDP	ROA	ESG	EIP	SDP
DCG	0.005*** (3.140)	0.523*** (2.914)	0.140** (2.261)	0.012*** (3.10)	0.005*** (3.228)	0.399** (2.333)	0.089** (2.048)	0.009*** (3.05)
Size					0.011*** (5.114)	1.804*** (9.314)	0.598*** (9.915)	0.048*** (13.32)
ATO					0.025*** (5.428)	0.531 (1.565)	0.366*** (3.783)	0.026*** (4.39)
TOP10					0.070*** (7.444)	1.406 (1.486)	1.528*** (5.981)	0.101*** (6.09)
GRO					0.017*** (4.407)	−0.637** (−2.075)	−0.075 (−0.888)	−0.008 (−1.47)
Indep					−0.000 (−0.261)	0.085** (2.419)	0.013 (1.482)	0.001** (2.53)
Dual					−0.011** (−2.205)	0.446 (1.049)	0.308*** (2.802)	0.019*** (2.66)
Balance					−0.009** (−2.132)	1.071** (2.400)	−0.331*** (−2.717)	−0.010 (−1.35)
Board					−0.004 (−0.658)	1.439** (2.099)	0.564*** (2.961)	0.042*** (3.48)
GS					0.001 (1.170)	0.028 (0.688)	−0.011 (−0.701)	−0.000 (−0.35)
AuditFee					−0.010*** (−3.904)	−0.393 (−1.366)	0.302*** (4.179)	0.012*** (2.80)
Employee					−0.011*** (−5.072)	−0.545*** (−3.115)	0.020 (0.402)	−0.004 (−1.22)
Constants	0.066*** (11.829)	73.332*** (140.823)	1.837*** (8.758)	0.389*** (29.28)	−0.003 (−0.108)	33.307*** (9.234)	−19.700*** (−20.587)	−1.121*** (−18.34)
Year Fe	Yes	Yes	Yes	Yes	YES	YES	YES	YES
Industry Fe	Yes	Yes	Yes	Yes	YES	YES	YES	YES
Obs	1,465	1,465	1,465	1,465	1,465	1,465	1,465	1,465
R^2^	0.138	0.117	0.230	0.194	0.262	0.258	0.619	0.623

Note: t-values are in parentheses; *, ** and *** passed the significance test at the level of 10%, 5% and 1% respectively, the same below.

### 4.3. Robustness tests

#### 4.3.1. Replacement of explanatory variables.

Referring to the methodologies of existing research, the digital transformation word frequency keyword library is reconstructed, and the quantity of word frequencies related to digital transformation uses Python as a replacement variable for the explanatory variable DCG. Columns (1) to (4) of Table 4 display the regression findings.

**Table 4:** Robustness test results 1.

Variable	Replacement of explanatory variables				Removing abnormal data			
(1)	(2)	(3)	(4)	(5)	(6)	(7)	(8)
ROA	ESG	EIP	SDP	ROA	ESG	EIP	SDP
*DCG*	0.001*** (2.855)	0.096*** (3.205)	0.027*** (3.162)	0.002*** (4.76)	0.005*** (2.998)	0.370** (2.092)	0.112** (2.456)	0.009*** (3.25)
Constants	−0.005 (−0.150)	33.228*** (9.213)	−19.714*** (−20.636)	−1.123*** (−18.40)	0.021 (0.677)	33.372*** (8.952)	−19.440*** (−19.780)	−1.103*** (−17.50)
Controls	Yes	Yes	Yes	Yes	Yes	Yes	Yes	Yes
Year Fe	Yes	Yes	Yes	Yes	Yes	Yes	Yes	Yes
Industry Fe	Yes	Yes	Yes	Yes	Yes	Yes	Yes	Yes
Obs	1,465	1,465	1,465	1,465	1,357	1,357	1,357	1,357
R^2^	0.260	0.259	0.620	0.625	0.268	0.261	0.621	0.622

#### 4.3.2. Exclusion of anomalous data.

The 2015 Chinese stock market crash had a notable effect on the stock exchange, which in turn was transmitted to enterprises and influenced digital transformation in some way. Therefore, based on earlier research, this study removes the enterprise samples from 2015 and conducts robustness tests on the empirical findings [56, 57]. Columns (5) to (8) of Table 4 display the regression findings.

#### 4.3.3. Adding control variables.

Other factors may influence energy enterprises’ sustainable development; thus, we add two control variables, the management expense ratio (Mshare) and the nature of property rights (SOE), for regression. The regression results are shown in Columns (1) through (4) of Table 5.

**Table 5:** Robustness test results 2.

Variable	Adding control variables				Explanatory variables lag one period			
(1)	(2)	(3)	(4)	(5)	(6)	(7)	(8)
ROA	ESG	EIP	SDP	ROA	ESG	EIP	SDP
*DCG*	0.004*** (2.767)	0.439** (2.538)	0.107** (2.466)	0.010*** (3.51)	0.004** (2.342)	0.464*** (2.625)	0.119** (2.513)	0.011*** (3.56)
Constants	−0.024 (−0.768)	32.810*** (9.039)	−19.905*** (−21.135)	−1.138*** (−18.78)	−0.046 (−1.348)	30.527*** (7.666)	−20.183*** (−19.419)	−1.173*** (−17.88)
Controls	Yes	Yes	Yes	Yes	Yes	Yes	Yes	Yes
Year Fe	Yes	Yes	Yes	Yes	Yes	Yes	Yes	Yes
Industry Fe	Yes	Yes	Yes	Yes	Yes	Yes	Yes	Yes
Obs	1,465	1,465	1,465	1,465	1,271	1,271	1,271	1,271
R^2^	0.279	0.261	0.623	0.629	0.259	0.266	0.624	0.632

#### 4.3.4. Lagged explanatory variables

Given that the extent of digital transformation can have a delayed impact, its effect on sustainable development performance will appear the following year. Therefore, we adapted the methodology of relevant research. To lag one period for the explanatory variable digital transformation (DCG) [58], Table 5, Columns [1] to [4] present the analytical outcomes of the regression model.

#### 4.3.5. Replacement of fixed effects.

To enhance the robustness of the regression results, this paper follows the practice of related scholars in conducting fixed effects regressions that control for year and control individuals to account for the impact of firm-level characteristics over time on the estimation results [59]. Table 6 reports the regression results, which remain significant after controlling for individual and year fixed effects, indicating that the findings are robust.

**Table 6:** Robustness test results 3.

Variable	Replacement of fixed effects			
(1)	(2)	(3)	(4)
ROA	ESG	EIP	SDP
*DCG*	0.004** (2.304)	0.332* (1.941)	0.076* (1.773)	0.005** (2.059)
Constants	−0.132 (−1.498)	65.196*** (7.166)	−6.357*** (−3.978)	−0.497*** (−3.898)
Controls	Yes	Yes	Yes	Yes
Year Fe	Yes	Yes	Yes	Yes
Stkcd Fe	Yes	Yes	Yes	Yes
Obs	1,465	1,465	1,465	1,465
R^2^	0.479	0.572	0.817	0.832

As shown in Tables 4, 5 and 6, every result from the robustness test aligns with the baseline test. These findings suggest that the conclusions of this study are robust.

### 4.4. Endogenous analysis

To address the problem of endogeneity, such as omitted variables, this study chooses instrumental variables, after which the regression results of endogeneity are tested using two-stage least squares. Although this paper selects control variables from multiple dimensions, it does not exclude the possibility that sustainable development performance may potentially be affected by multiple factors; consequently, this work cites other research studies. The instrumental variable approach is used to address the endogeneity of the empirical evidence. Two variables are selected: the digital transformation of energy industries, less the average water value of energy enterprises’ digital transformation, broken down by year and industry, which is denoted as IV1; the second is the digital transformation that is one period behind schedule and denoted as IV2, after which the two-stage least squares (2SLS) method is used for testing. The first-stage regression findings are presented in Table 7 Column (1). The findings indicate that the instrumental variables and digital transformation (DCG) are remarkably favorable and pass the instrumental variables validity test. Compared with the C-D Wald F statistic and the K-P Wald rk F statistic, the 10% critical value for the Stock–Yogo test is substantially smaller, with values of 2,719.393 and 3,868.656, respectively, confirming the absence of weak instrument bias in the simultaneous equations framework. The K-P rk LM statistic is significant at the 1% level, indicating there is no underidentification problem, and the Hansen J statistic has a value of 0.3468 > 0.1 and is not significant, indicating there is no issue with overidentification. The outcomes of the second-stage regression are displayed in Table 7, Columns (2) through (5). After considering the absence of endogenous interference problems, the sustainability performance of energy businesses is still significantly positively affected by digital transformation, demonstrating congruence with the primary econometric model’s outputs.

**Table 7:** Endogeneity test results.

Variable	(1)	(2)	(3)	(4)	(5)
DCG	ROA	ESG	EIP	SDP
IV1	0.8818*** (52.63)				
IV2	0.2004*** (11.21)				
DCG		0.0050*** (3.00)	0.3578* (1.94)	0.1513*** (2.87)	0.0115*** (3.56)
controls	YES	YES	YES	YES	YES
Year FE	YES	YES	YES	YES	YES
Industry FE	YES	YES	YES	YES	YES
Constants	0.0118 (0.04)	−0.0561* (−1.83)	35.7123*** (9.94)	−19.4566*** (−20.31)	−1.0950*** (−17.95)
C-D Wald F statistic	2719.393				
K-P Wald rk F statistic	3868.656				
K-P rk LM statistic	412.644				
Hansen J statistic	0.3468				
Obs	1,271	1,271	1,271	1,271	1,271
R^2^	0.812	0.179	0.168	0.517	0.546

## 5. Further analyses

### 5.1. Mechanism effect tests

To test whether financing constraints and resource allocation efficiency play a moderating role in the relationship between sustainable development performance and the degree of digital transformation, this paper adopts the method of Wen and Ye [60] and uses stepwise regression to create the following mediating effect model based on Model (1):


$$\begin{array}{c} KZ_{i,t}\;/GTEP_{i,t}\;=\;\beta_{0}+\;\beta_{1}DCG_{i,t}\;+\;\beta_{2}Control_{i,t}\;+\;Ind_{i}\;+\;Year_{t}\;+\;\varepsilon_{i,t} \end{array}$$ (2)

$$\begin{array}{c} ROA_{i,t}\;/ESG_{i,t}\;/EIP_{i,t}\;/SDP_{i,t}\;=\;\gamma_{0}+\;\gamma_{1}DCG_{i,t}\;+\;\gamma_{2}KZ_{i,t}\;+\;\gamma_{3}Control_{i,t}\;+Ind_{i}\;+\;Year_{t}\;+\;\varepsilon_{i,t} \end{array}$$ (3)

$$\begin{array}{c} ROA_{i,t}\;/ESG_{i,t}\;/EIP_{i,t}\;/SDP_{i,t}\;=\;\gamma_{0}+\;\gamma_{1}DCG_{i,t}\;+\;\gamma_{2}TFP_{i,t}\;+\;\gamma_{3}Control_{i,t}\;+\;Ind_{i}\;+\;Year_{t}\;+\;\varepsilon_{i,t} \end{array}$$ (4)

Where *β*_*1*_ in Model (2) represents the influence of green financial development on financing constraints and resource allocation efficiency in energy firms; and the interaction term (*β*_*1*_ × *γ*_*2*_) between *β*_*1*_ in Model (2) and *γ*_*2*_ in Models (3) and (4) symbolizes the mediating influence of financing constraints and resource allocation efficiency.

#### 5.1.1. Financing constraint mechanisms.

The findings of the mediation effect test regarding financial constraints are displayed in Table 8, Column (1), which presents the results of digital transformation’s effect on financial limitations, showing a DCG regression coefficient of −1.07 that is statistically significant at the 10% level. This indicates a significant negative relationship, suggesting that digital transformation contributes to reducing the financial constraints faced by energy organizations. For the KZ index, the regression coefficients in Columns (2) to (5) are −0.16, −0.429, −0.033, and −0.007, respectively, which are statistically significant. These results imply that financing constraints have a negative effect on the financial performance of energy firms, as do environmental and social governance performance, innovation performance, and sustainability performance. Thus, increased financing constraints hinder improvements in energy enterprises’ sustainable development performance. The DCG coefficients in Columns (2) to (5) are 0.003, 0.346, 0.086, and 0.008, respectively, which are also statistically significant and positive. Notably, the overall effect coefficient of digital transformation is smaller than that in Table 3’s baseline regression, demonstrating that financial constraints play a mediating role; thus, Hypothesis H2 is validated.

**Table 8:** Results of the mediation effect test for financial constraints.

Variable	(1)	(2)	(3)	(4)	(5)
KZ	ROA	ESG	EIP	SDP
DCG	−0.107* (−1.742)	0.003*** (2.61)	0.346** (2.06)	0.086* (1.96)	0.008*** (2.79)
KZ		−0.016*** (−25.17)	−0.492*** (−7.05)	−0.033* (−1.69)	−0.007*** (−5.64)
constant	−0.722 (−0.546)	−0.015 (−0.64)	32.952*** (9.20)	−19.724*** (−20.66)	−1.126*** (−18.65)
Controls	Yes	Yes	Yes	Yes	Yes
Year Fe	Yes	Yes	Yes	Yes	Yes
Industry Fe	Yes	Yes	Yes	Yes	Yes
Obs	1,465	1,465	1,465	1,465	1,465
R2	0.272	0.565	0.283	0.620	0.632

#### 5.1.2. Resource allocation efficiency mechanisms.

The results of the mediation analysis evaluating resource allocation pathways are systematically presented in Table 9. Column (1) of Table 9 demonstrates that digital transformation has a robust positive influence on energy firms’ resource optimization (DCG *β* = 0.055, p < 0.01). Complementary evidence in Table 9, Column (5) reveals dual mediation effects, with DCG and TFP coefficients measuring 0.008 (p < 0.01) and 0.012 (p < 0.10), respectively. This empirical evidence confirms the partial mediating role of resource allocation efficiency in linking digital transformation to corporate sustainability outcomes, thereby corroborating Hypothesis H3. Furthermore, Columns (2) to (4) of Table 9 reveal significantly positive coefficients for both DCG and TFP, suggesting that digital transformation enhances energy firms’ financial performance, environmental and social governance performance, and innovation performance through improved resource allocation efficiency.

**Table 9:** Resource allocation efficiency mediation affects test results.

Variable	(1)	(2)	(3)	(4)	(5)
TFP	ROA	ESG	EIP	SDP
DCG	0.055*** (4.670)	0.005*** (3.00)	0.395** (2.28)	0.074* (1.67)	0.008*** (2.79)
TFP		0.006* (1.75)	1.262*** (5.03)	0.283*** (2.85)	0.012* (1.92)
constant	−6.059*** (−21.409)	0.032 (0.86)	49.723*** (14.05)	−17.985*** (−16.05)	−1.050*** (−14.98)
Controls	Yes	Yes	Yes	Yes	Yes
Year Fe	Yes	Yes	Yes	Yes	Yes
Industry Fe	Yes	Yes	Yes	Yes	Yes
Obs	1,465	1,465	1,465	1,465	1,465
R^2^	0.901	0.264	0.230	0.621	0.624

## 6. Heterogeneity analysis

### 6.1. Heterogeneity in firm size

The differences in organizational strategies and structures among companies of varying sizes may influence the effect of digital transformation on energy organizations’ sustainability performance. Therefore, this study examines heterogeneity by using the median logarithm of a firm’s total assets and dividing the sample into two groups—large enterprises and small and medium-sized firms. Table 10, Column 1 displays the regression results. Large energy firms’ financial performance is positively impacted by digital transformation because they are concerned with financial performance and tend to use digital transformation to enhance financial performance. As shown in Columns (2) through (4), digital transformation has limited effects on environmental and social governance performance, innovation performance, and sustainability performance of large-scale energy companies. Columns (6)– (8) reveal statistically meaningful enhancements in ESG metrics (p < 0.05), innovation outcomes (p < 0.1), and sustainability indicators (p < 0.01) among SME energy enterprises undergoing digital transformation. This pattern aligns with these firms’ strategic prioritization of market penetration and scalable development, where technological adoption serves as a catalyst for growth. Sustainability performance can be improved through digital transformation to achieve rapid growth goals. Although they are profitable, large energy businesses typically do not use digital transformation to improve sustainability performance because they lack flexibility.

**Table 10:** Results of firm size heterogeneity test.

Variable	Large-scale firms				Small and medium-sized firms			
(1)	(2)	(3)	(4)	(5)	(6)	(7)	(8)
*ROA*	*ESG*	*EIP*	*SDP*	*ROA*	*ESG*	*EIP*	*SDP*
*DCG*	0.004** (2.24)	0.051 (0.18)	0.086 (1.32)	0.005 (1.25)	0.003 (1.32)	0.465** (2.23)	0.107* (1.76)	0.010*** (2.66)
Constants	0.077** (2.43)	21.802*** (3.47)	−22.236*** (−15.15)	−1.365*** (−13.87)	−0.024 (−0.28)	52.081*** (6.31)	−11.728*** (−5.38)	−0.513*** (−3.75)
Controls	Yes	Yes	Yes	Yes	Yes	Yes	Yes	Yes
Year Fe	Yes	Yes	Yes	Yes	Yes	Yes	Yes	Yes
Industry Fe	Yes	Yes	Yes	Yes	Yes	Yes	Yes	Yes
Obs	735	735	735	735	730	730	730	730
R^2^	0.270	0.233	0.678	0.660	0.299	0.262	0.378	0.377

### 6.2. Heterogeneity of enterprise industry chain links

Owing to variations in the locations and functions of energy companies across the supply chain (e.g., upstream vs. downstream), digital transformation may differentially affect the sustainable development performance of upstream versus downstream energy enterprises. To investigate this, energy firms were categorized into upstream and downstream groups on the basis of their positions in the industry chain: upstream energy enterprises are involved in coal and petroleum extraction, with industry codes B06, B07, and B11, whereas downstream energy enterprises include petroleum processing and electricity and gas supply firms, with industry codes C25, D44, D45, and D46. Table 11 presents the regression results for the two sample groups; regressions (1 ,2 ), and (4) indicate that digital transformation affects the financial performance, environmental and social governance performance, and sustainability performance of upstream energy enterprises. Governance performance and sustainable development performance are not significant because the upstream energy enterprises represented by the coal and oil extraction and processing industries are more dependent on fossil energy raw materials. Therefore, the performance of upstream energy companies in terms of sustainable development is not significantly impacted by digital transformation. According to Column (3), digital transformation notably improves the innovation performance of upstream energy firms because digital transformation allows upstream energy enterprises to better manage and operate complex production systems through artificial intelligence technology to predict the failure of equipment and maintenance plans, which also increases the inventiveness of investments in research and development and improves the efficiency of extraction. The sustainability performance of downstream energy companies is significantly improved by digital transformation, represented by electricity and gas companies, which are directly exposed to market demands and are more sensitive to environmental standards. Thus, they are more receptive to the benefits of digital transformation and are more driven to invest in creating clean energy goods that the public demands.

**Table 11:** Heterogeneity test results of enterprise industry chain links.

Variable	Upstream enterprise				downstream enterprise			
(1)	(2)	(3)	(4)	(5)	(6)	(7)	(8)
*ROA*	*ESG*	*EIP*	*SDP*	*ROA*	*ESG*	*EIP*	*SDP*
*DCG*	0.005 (1.64)	−0.177 (−0.52)	0.148* (1.71)	0.007 (1.23)	0.005*** (2.71)	0.487** (2.51)	0.038 (0.75)	0.006** (2.02)
Constants	−0.204*** (−3.46)	12.622* (1.75)	−18.646*** (−10.25)	−1.253*** (−10.24)	0.043 (1.29)	39.460*** (10.55)	−21.237*** (−18.23)	−1.154*** (−15.54)
Controls	Yes	Yes	Yes	Yes	Yes	Yes	Yes	Yes
Year Fe	Yes	Yes	Yes	Yes	Yes	Yes	Yes	Yes
Industry Fe	Yes	Yes	Yes	Yes	Yes	Yes	Yes	Yes
Obs	457	457	457	457	1,008	1,008	1,008	1,008
R^2^	0.469	0.300	0.678	0.690	0.268	0.284	0.582	0.596

## 7. Conclusions and recommendations

### 7.1. Conclusions of the study

This empirical analysis examines the sustainability implications of digital transformation through panel data from China’s publicly traded energy sector (2011–2023). Utilizing a fixed-effects panel regression framework, the research reveals three critical patterns:

First, digital transformation helps energy companies perform better in terms of sustainable development, enhancing their energy financial performance, environmental and social governance performance, and innovation performance.

Second, digital transformation improves energy firms’ sustainability performance and subdimensional performance in terms of energy financial performance, environmental social governance performance and innovative performance by reducing financial barriers and increasing the effectiveness of resource allocation.

Third, for large-scale enterprises, digital transformation affects the financial performance of energy companies more significantly, whereas for small-scale enterprises, digital transformation influences the performance of socioenvironmental governance, financial performance, and sustainable development performance more significantly. Additionally, digital transformation impacts innovation performance more significantly. The financial performance of upstream and downstream energy enterprises is more significantly improved by digital transformation, social and environmental governance performance, and sustainability performance.

### 7.2. Recommendations

(1) Energy company managers must actively introduce digital technologies. Currently, most energy companies have implemented little to no digital transformation, with enterprise management concerned that carrying out digital transformation will not yield the expected returns. This study confirms that digital transformation has a favorable impact on energy companies’ sustainable development performance; these findings should help energy enterprise management alleviate these concerns and apply digital technology to the manufacturing processes to achieve economic and environmental benefits. Additionally, digital transformation cannot be achieved overnight, and enterprise managers must not give up in the middle of the process. Following Kang Tang [61], energy companies can establish a performance evaluation system using the five dimensions of the balanced scorecard approach: financial, customer, internal operations, social responsibility, and learning and growth.
(2) Energy managers must develop diversified financing channels and rationally allocate resources. Digital transformation has been shown to improve energy companies’ sustainable development performance by reducing funding limitations and increasing the effectiveness of resource allocation. Therefore, energy enterprises should open a variety of financing channels to effectively alleviate the problem of project funding limitations, optimize available resource use, and avoid the dangers of short-term funding for long-term investments. Additionally, energy enterprises can enhance business performance in terms of sustainable development by improving resource allocation efficiency. For example, energy companies can adopt intelligent equipment, engage in the real-time collection of production data and information technology use and scientifically allocate resources, thereby reducing pollution emissions.
(3) Energy companies must focus on improving sustainable development performance and persevere. Research has found that implementing digital transformation can significantly enhance the sustainable development performance of energy companies, yet digitally transforming businesses requires continuous change and innovation. Therefore, energy enterprises should continue to explore new digital technologies, continuously improve and optimize their production processes and management methods, and enhance their sustainable development through digital transformation. For example, energy companies should embed digital development into their sustainable development strategy, increase their core competitiveness and innovate new products through digital transformation, improve financial performance while reducing the adverse effects on the ecosystem, and realize the sustainable development goal of a harmonious coexistence between the enterprise and the natural environment.
(4) The government should consider providing subsidies for the digital transformation of firms and formulate relevant policies to guide and incentivize energy enterprises to carry out digital transformation. The government can provide subsidies or tax incentives to energy enterprises to encourage them to implement digital transformation. Additionally, the government can actively develop an innovative platform to reduce the cost of introducing digital technologies for energy enterprises by integrating the knowledge resources of universities, enterprises, and R&D organizations, thereby better realizing sustainable development.

## 8. Research contributions

### 8.1. Theoretical implications

This investigation advances scholarly discourse through three principal innovations:

(1) Research on digital transformation in energy enterprises should be enriched. First, while the literature has not reached a consensus on the impact of digital transformation on corporate sustainability performance, with three prevailing perspectives suggesting positive, negative, or curvilinear effects—empirical research remains limited, particularly those employing quantitative methodologies. This study systematically examines the drivers, pathways, and mediating mechanisms through which digital transformation influences sustainability performance in energy firms, reexamining this relationship and providing empirical evidence to clarify the economic implications of organizational digitalization.
(2) Expanding the research scope of sustainability performance. Building on foundational theoretical frameworks, this study advances sustainability performance measurement by establishing a tripartite dimensional structure encompassing economic performance, environmental‒social governance (ESG), and innovation capabilities. It further constructs corresponding indicators and investigates their determinants and underlying mechanisms, offering a robust empirical foundation for future inquiries into multidimensional sustainability outcomes.

### 8.2. Managerial implications

(1) This study provides an academic foundation for governments to formulate targeted and efficient policies. As energy sustainability has become a critical performance metric for high-quality corporate development, sustainability outcomes are shaped by multifaceted internal and external factors. Digital transformation, while serving as a strategic approach to enhance organizational capabilities and streamline operational processes, exerts complex influences on sustainability performance. Through systematic analysis of energy enterprises’ operational realities, this study proposes actionable regulatory interventions and refined management systems to standardize pollution emission practices. These measures could help reduce the opportunity costs of digital adoption, accelerate energy firms’ digital transition, and ultimately foster coordinated development across economic, social, environmental, and innovation dimensions.
(2) This study offers empirical evidence to guide energy enterprises in optimizing digital transformation. By investigating how digital transformation affects sustainability performance through financing constraints and resource allocation efficiency, this research elucidates the mechanisms through which digitalization empowers sustainable development. The findings provide targeted operational frameworks to facilitate deeper integration between core energy operations and digital technologies. Specifically, the analysis of mediating pathways offers practical insights for enterprises to alleviate financing bottlenecks in digital adoption, enhance resource allocation precision through data-driven decision-making, and balance technological investments with sustainability imperatives. Such evidence-based guidance supports energy firms in achieving high-quality development while navigating the challenges of digital transition.

## 9. Limitations and further research

This article examines how digital transformation affects energy companies’ sustainable development performance and studies the mediating role of financing constraints and resource allocation efficiency, enriching existing research. However, the research procedure still has limitations. First, although academics typically employ an approach to quantify digital transformation based on word frequency as a proxy variable, with the continuous deepening of research, it is possible to improve measurement techniques for digital transformation. Second, since only Chinese A-share listed companies provided the data for this study, the research results may not represent all firms, particularly nonlisted businesses and small and medium-sized businesses. Additionally, this study may have overlooked variations in digital demand and response tactics under different property rights, as it did not fully account for the heterogeneity of property rights. Finally, the findings of this study might not be applicable to people from various cultural and geographic backgrounds, as the study only examines the Chinese market.

On the basis of the aforementioned limitations, future research will focus on the following directions:

First, we aim to refine the measurement of digital transformation. While existing studies predominantly rely on textual analysis (e.g., keyword frequency) to assess digital transformation, this approach has inherent limitations, such as oversimplification and context insensitivity. Our subsequent work will explore more precise, multidimensional metrics—potentially integrating structured operational data (e.g., R&D expenditure allocation, technology adoption timelines) with unstructured behavioral indicators (e.g., executive strategic narratives)—to better capture the heterogeneity of corporate digitalization.

Second, we investigate how varying degrees of digital transformation influence corporate performance. Building on this, we intend to develop a comprehensive performance evaluation framework that systematically accounts for both technical determinants (e.g., data infrastructure scalability) and nontechnical factors (e.g., organizational culture, and scientific and technological talent). This framework will enable tailored strategies for enterprises at different levels of digital maturity.

Finally, expanding beyond the Chinese context, we will conduct cross-national comparative studies to examine how institutional, cultural, and market-specific factors shape digital transformation pathways and sustainability outcomes. Such insights will provide globally relevant policy and managerial implications, particularly for emerging economies navigating the dual challenges of technological disruption and sustainable development.

## Supporting information

S1 Data (XLSX)
